# Insulin-like peptide 3 (INSL3) in congenital hypogonadotrophic hypogonadism (CHH) in boys with delayed puberty and adult men

**DOI:** 10.3389/fendo.2022.1076984

**Published:** 2022-11-29

**Authors:** Ali Abbara, Kanyada Koysombat, Maria Phylactou, Pei Chia Eng, Sophie Clarke, Alexander N. Comninos, Lisa Yang, Chioma Izzi-Engbeaya, Simon Hanassab, Neil Smith, Channa N. Jayasena, Cheng Xu, Richard Quinton, Nelly Pitteloud, Gerhard Binder, Ravinder Anand-Ivell, Richard Ivell, Waljit S. Dhillo

**Affiliations:** ^1^ Section of Investigative Medicine, Imperial College London, London, United Kingdom; ^2^ Department of Endocrinology, Imperial College Healthcare National Health Service (NHS) Trust, London, United Kingdom; ^3^ Department of Computing, Imperial College London, London, United Kingdom; ^4^ Kallmann Syndrome Patient Support Group, London, United Kingdom; ^5^ Service of Endocrinology, Diabetology & Metabolism, Centre Hospitalier Universitaire Vaudois (CHUV), Lausanne, Switzerland; ^6^ Faculty of Biology and Medicine, University of Lausanne, Lausanne, Switzerland; ^7^ Translational & Clinical Research Institute, University of Newcastle-upon-Tyne, Newcastle, United Kingdom; ^8^ The Newcastle upon Tyne Hospitals National Health Service (NHS) Foundation Trust, Newcastle, United Kingdom; ^9^ Department of Paediatric Endocrinology, University Children’s Hospital, Tübingen, Germany; ^10^ School of Biosciences, University of Nottingham, Nottingham, United Kingdom

**Keywords:** insulin-like peptide 3, INSL3, inhibin B, congenital hypogonadotrophic hypogonadism, CHH, constitutional delay of growth and puberty, CDGP, delayed puberty

## Abstract

**Background:**

Delayed puberty in males is almost invariably associated with constitutional delay of growth and puberty (CDGP) or congenital hypogonadotrophic hypogonadism (CHH). Establishing the cause at presentation is challenging, with “red flag” features of CHH commonly overlooked. Thus, several markers have been evaluated in both the basal state or after stimulation e.g. with gonadotrophin releasing hormone agonist (GnRHa).

Insulin-like peptide 3 (INSL3) is a constitutive secretory product of Leydig cells and thus a possible candidate marker, but there have been limited data examining its role in distinguishing CDGP from CHH. In this manuscript, we assess INSL3 and inhibin B (INB) in two cohorts: 1. Adolescent boys with delayed puberty due to CDGP or CHH and 2. Adult men, both eugonadal and having CHH.

**Materials and methods:**

Retrospective cohort studies of 60 boys with CDGP or CHH, as well as 44 adult men who were either eugonadal or had CHH, in whom INSL3, INB, testosterone and gonadotrophins were measured.

*Cohort 1:* Boys with delayed puberty aged 13-17 years (51 with CDGP and 9 with CHH) who had GnRHa stimulation (subcutaneous triptorelin 100mcg), previously reported with respect to INB.

*Cohort 2:* Adult cohort of 44 men (22 eugonadal men and 22 men with CHH), previously reported with respect to gonadotrophin responses to kisspeptin-54.

**Results:**

Median INSL3 was higher in boys with CDGP than CHH (0.35 vs 0.15 ng/ml; *p*=0.0002). Similarly, in adult men, median INSL3 was higher in eugonadal men than CHH (1.08 vs 0.05 ng/ml; *p*<0.0001). However, INSL3 more accurately differentiated CHH in adult men than in boys with delayed puberty (auROC with 95% CI in *adult men:* 100%, 100-100%; *boys with delayed puberty:* 86.7%, 77.7-95.7%).

Median INB was higher in boys with CDGP than CHH (182 vs 59 pg/ml; *p*<0.0001). Likewise, in adult men, median INB was higher in eugonadal men than CHH (170 vs 36.5 pg/ml; *p*<0.0001). INB performed better than INSL3 in differentiating CHH in boys with delayed puberty (auROC 98.5%, 95.9-100%), than in adult men (auROC 93.9%, 87.2-100%).

**Conclusion:**

INSL3 better identifies CHH in adult men, whereas INB better identifies CHH in boys with delayed puberty.

## Introduction

Pubertal disorders are common and are associated with considerable psychosocial impact for those affected and their families (1, 2). Delayed pubertal onset is defined as the absence of testicular enlargement to a volume of ≥4 ml at an age that is 2-2.5 standard deviations (SD) later than the population mean, typically defined as age ≥14 years in boys (3).

The overwhelming majority of boys (95%) with delayed puberty will have inappropriately normal or low levels of gonadotrophins in the context of low sex steroids due to permanent or transient gonadotrophin deficiency (4). Of these, the vast majority of younger adolescents (60-80%) will have constitutional delay of growth and puberty (CDGP) and will spontaneously proceed through normal puberty but later than their peers (5–7). However, around 10% of younger adolescent boys with delayed puberty will have congenital hypogonadotrophic hypogonadism (CHH), rising to over 90% by 18-20 years (8). CHH is a genetic trait causing impaired hypothalamic gonadotrophin releasing hormone (GnRH) neuronal migration, leading to lack or deficient number of GnRH neurons; or function, as result of disturbed secretion or action of GnRH, or both (9). A further 10-20% have functional causes of HH such as acute or chronic illness, insufficient energy availability or side-effects of medications e.g., glucocorticoids (4).

Distinguishing CDGP from CHH poses a major diagnostic challenge in boys with delayed puberty particularly owing to the overlapping clinical features and similar hormonal profiles (10). With median age of effective treatment of CHH males remaining stubbornly high at 18-19 years – principally due to confusion with CDGP – it is clear that clinicians find this a difficult distinction. The accurate and timely distinction between CDGP and CHH is important for appropriate counselling and treatment i.e. conservative management or sex-steroid treatment for psychological distress in CDGP versus a greater consideration towards GnRH pump/gonadotrophin treatment in those with CHH with a view to address sexual function, bone, metabolic and psychological health (11). Although the majority of CHH males harbor phenotypic “red flags” (anosmia, cryptorchidism, deafness, clefting, or other developmental anomaly), their significance is commonly not appreciated (12).

Leydig and Sertoli cell markers, such as insulin-like peptide 3 (INSL3) and inhibin B (INB) respectively, have been evaluated as potential markers to differentiate CDGP and CHH. INB is a member of the transforming growth factor β (TGFβ) superfamily. INB is a glycoprotein heterodimer comprised of an inhibin alpha-subunit and an inhibin beta B-subunit, which is secreted by and is reflective of the number and function of Sertoli cells (13). INB peaks transiently during the mini-puberty at around 2-4 months after birth, and then decreases during childhood prepubertally before increasing again during puberty (14). The INB surge in male puberty may be appreciable as early as 9 years old and thus precedes the appearance of measurable LH surge (15). In prepubertal boys, INB reflects predominantly Sertoli cell mass and function, while in adulthood, germ cells are major determinants of the α-subunit and INB production. Consequently, in adulthood INB closely reflects testicular mass including germ cells as well as Sertoli cells, and thus spermatogenic capability, and thus could be reduced in men with CHH. INB negatively regulates FSH secretion from the anterior pituitary gland such that the level of INB is inversely correlated with FSH levels in adult men (13). Most studies to date largely investigated the potential use of INB to differentiate CDGP and CHH, whilst fewer studies have evaluated INSL3 (16–18). INB is a marker of mature Sertoli cells (19), whilst INSL3 reflects the number, and degree of differentiation of Leydig cells (20).

INSL3 has been shown to demonstrate age-dependent dynamics from birth to adulthood (21). Fetal INSL3 plays a pivotal role in the initial transabdominal testicular descent (22). Significantly higher levels of INSL3 were found in serum from 3-month-old boys compared to older prepubertal boys reflecting the transient postnatal gonadotrophin surge or ‘mini-puberty’ (21). Serum INSL3 levels increment again during puberty (23) and remain high throughout adulthood before declining with age (24). Pathologies affecting the hypothalamo-pituitary-gonadal (HPG) axis including CHH and primary testicular disorders including Klinefelter syndrome, cryptorchidism and anorchidism are all associated with lower levels of INSL3 (25). Unlike testosterone, INSL3 is not subject to episodic fluctuations inherent to the feedback loop in the HPG axis (26), thus making INSL3 an emerging biomarker of disorders affecting the male HPG axis.

In this study, we evaluated the performance of the testicular markers INSL3 and INB in two cohorts; first in boys with delayed puberty due to either CHH or CDGP, and second in adult men with either CHH and eugonadal controls to determine the role of INSL3 and INB as biomarkers in CHH and delayed puberty.

## Materials and methods

### Ethical approval

Ethical approval for the study comparing eugonadal men and men with CHH was granted by the West London Research Ethics Committee, London, United Kingdom (UK) (reference: 12/LO/0507). Ethical approval for the study comparing boys with CDGP and boys with CHH was granted by the Ethical Committee of the Medical Faculty of the University of Tübingen, Germany. Written informed consent was provided by all participants. Both studies were conducted in accordance with the Declaration of Helsinki.

### Participants

#### Boys with delayed puberty

Participants with delayed puberty due to either CHH or CDGP were selected from boys who presented with delayed puberty to the Department for Paediatric Endocrinology of the University Children’s Hospital Tübingen between the years 2009 and 2013. A diagnosis of CDGP was made at 12-18 months review if the testicular volume (TV) reaches ≥8ml, if TV did not reach a volume of ≥5ml then CHH was assumed at 24 months follow-up. All the boys included in the analysis had TV of ≥8ml at 12-18 months (CDGP) or <5ml at 24 months (CHH). This cohort has been previously reported in a study determining the discriminatory power of INB and luteinising hormone (LH) vs GnRH agonist (GnRHa) test (27). Participants fulfilled the following criteria: age 13-17 years, demonstrated clinical phenotype of delayed puberty at the time of testing including TV <4ml. Boys with delayed puberty were either at Tanner stage G1 with TV ≤3ml or had some evidence of pubertal progress (boys with Tanner stage G2 with TV 3.5-4ml) (27). Participants with hypopituitarism, functional HH, or already diagnosed with CHH, were excluded from this study. All participants underwent detailed medical assessment including full medical history and physical examination by a paediatric endocrinologist for syndromic features and clinical signs of hypopituitarism or other disorders that can cause functional HH. Tanner staging system was used for pubertal staging, Prader orchidometer was used to measure TV and olfactory function quantified using Sniffin’ Sticks (Burghart Messtechnik GmbH, Wedel, Germany) in participants suspected to have CHH. Bone age was determined by obtaining left hand radiographs which were reviewed by three experienced paediatric endocrinologists using the Greulich and Pyle radiographic atlas. All participants had measurement of basal serum levels of LH, follicle stimulating hormone (FSH), INSL3, testosterone and INB. A single subcutaneous injection of 100mcg of triptorelin acetate (DECAPEPTYL^®^ IVF 0.1 mg/ml; Ferring Arzneimittel, Kiel, Germany) was administered followed by serial blood sampling at 4 and 24 hours for serum LH, FSH and testosterone.

#### Adults with CHH or eugonadal controls

Participants in the CHH and eugonadal men cohort were recruited through newspaper advertisements, endocrine clinics, and *via* the CHH online community. In all, 22 adult eugonadal men and 22 men with CHH were recruited. This cohort was previously reported with regards to their responses to kisspeptin-54 (28). All participants underwent detailed medical assessment including full medical history and physical examination. The following criteria were fulfilled by eugonadal men: age 18-35 years, BMI 18-30 kg/m^2^, absence of significant co-morbidities, systemic disease, regular therapeutic or recreational drug use and normal clinical and biochemical reproductive function. Participants with CHH were defined as having hypogonadotrophic hypogonadism with a history of incomplete progression through puberty by the age of 18 years. Men with CHH underwent further examination including TV measurement using a Prader orchidometer, evaluation for syndromic features and clinical signs of hypopituitarism and olfactory function quantified using the 40-item University of Pennsylvania Smell Identification Test (UP-SIT). All participants had measurement of basal serum levels of LH, FSH, INSL3, testosterone and INB. CHH men also had genetic testing to identify genes implicated in the aetiology of CHH. Four adult men with CHH had a pathogenic or likely pathogenic variant identified in the genes: *ANOS1*, *FGFR1*, *PROKR2*, and *SEMA3A* as previously detailed by Abbara et al. (28). Participants with CHH were asked to discontinue testosterone gel for at least 1 week prior to participation in the study. Study visits for those on longer acting intramuscular testosterone preparations were conducted when the participants’ testosterone levels were at trough level prior to the next injection to minimise disruption to their treatment. Participants with CHH did not receive gonadotrophin therapy for at least 6 weeks prior to participation in the study. Participants attended two study visits with a washout period of at least 1 week. Kisspeptin-54 (6.4 nmol/kg; Bachem AG, Liverpool, UK) or GnRH (Gonadorelin 100mcg; Intrapharm Laboratories Ltd., Maidenhead, Berks, UK) were administered intravenously as a single bolus at each study visit in random order after a 30-minute period of baseline blood-sampling. INB was measured at baseline and serial blood-sampling was performed at 15-minute intervals for 6 hours to measure serum LH, FSH and testosterone levels.

### Hormone assays used in the paediatric CHH and CDGP cohort

INB (solid-phase sandwich enzyme-linked immunosorbent assay (ELISA), Beckman Coulter; intra-assay and inter-assay coefficient of variation 5.7 and 6.8% respectively). Analytical sensitivity was 2.6 pg/ml. INSL3 was measured using a well-validated and specific time-resolved fluorescent immunoassay (24) modified for high sensitivity (29). Intra- and inter-assay coefficients of variation were <3% and <10% across the range, with an analytical sensitivity of 0.01 ng/ml. LH (chemiluminescence assay, Siemens Healthcare Diagnostics GmbH; inter-assay coefficient of variation <4%). Analytical sensitivity 0.1 mU/L. FSH (chemiluminescence assay, Siemens Healthcare Diagnostics GmbH; inter-assay coefficient <8%). Analytical sensitivity 0.2 mU/L. Testosterone (radioimmunoassay, Siemens Medical Products; inter-assay and intra-assay coefficient of variation 9.3 and 6.4% respectively). Analytical sensitivity was 0.14nmol/L.

### Hormone assays used in the CHH and eugonadal men cohort

INB (solid-phase sandwich ELISA, intra-assay and inter-assay coefficient of variation 6.6 and 5.6% respectively). Reference range for eugonadal men: 25-325 ng/L. Analytical sensitivity 2.91 pg/ml. INSL3 was measured as above. Values <0.005 ng/ml were set at 0.004 ng/ml for analysis. LH (chemiluminescence assay, Abbott Diagnostics; intra-assay and inter-assay coefficient of variation 2.7 and 4.1% respectively). Reference range for eugonadal men: 2.0-12.0 IU/L. Analytical sensitivity 0.03 IU/L. FSH (chemiluminescence assay, Abbott Diagnostics; intra-assay and inter-assay coefficient of variation 3.0 and 4.1% respectively). Reference range for eugonadal men: 1.7-8.0 IU/L. Analytical sensitivity 0.05 IU/L. Testosterone (chemiluminescence assay, Abbott Diagnostics; intra-assay and inter-assay coefficient of variation 2.8 and 4.2% respectively). Reference range for eugonadal men, total testosterone: 10.0-30.0 nmol/L. Analytical sensitivity 0.05 nmol/L.

### Statistical methods

Statistical analyses were conducted using GraphPad Prism version 9.0. Normality was determined by the D’Agostino-Pearson test. Parametric variables were reported as mean ± SD and compared using the unpaired *t*-test. Non-parametric variables were reported as median (interquartile range) and compared using the Mann-Whitney U test. Discriminatory potential was assessed by receiver operated characteristic (ROC) analysis. Proportions were compared by linear regression. *P* values <0.05 were regarded as statistically significant.

## Results

### Baseline characteristics

The clinical characteristics of the 51 boys with CDGP and 9 boys with CHH [previously described by Binder et al. (27)], as well as the 22 eugonadal men, and 22 men with CHH [previously described by Abbara et al. (28)] are presented in **Supplementary Table 1**. In boys with delayed puberty, those with CHH had significantly higher BMI and were taller compared to those with CDGP. TV and bone age were lower in the CHH group compared to those with CDGP. Men with CHH were recruited at any age and were older and had higher BMI than eugonadal men.

### INSL3 in delayed puberty

**Figure 1:**
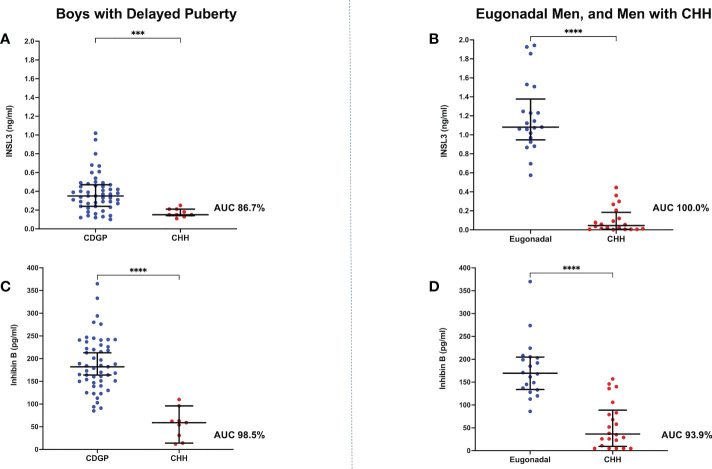
**(A)** Scattergram (median, IQR) of basal INSL3 (ng/ml) in boys with CDGP in blue and boys with CHH in red. Groups were compared using the Mann-Whitney U test (***p value 0.0002). auROC of 86.7%. **(B)** Scattergram (median, IQR) of basal INSL3 (ng/ml) in eugonadal men in blue and men with CHH in red. Groups were compared using the Mann-Whitney U test (****p value <0.0001). auROC of 100%. **(C)** Scattergram (median, IQR) of basal INB (pg/ml) in boys with CDGP in blue and boys with CHH in red. Groups were compared using the Mann-Whitney U test (****p value <0.0001). auROC of 98.5%. **(D)** Scattergram (median, IQR) of basal INB (pg/ml) in eugonadal men in blue and men with CHH in red. Groups were compared using the Mann-Whitney U test (****p value <0.0001). auROC of 93.9%.

The median (IQR) basal INSL3 was 0.35 ng/ml (0.24, 0.47) in boys with CDGP and 0.15 ng/ml (0.14, 0.21) in boys with CHH (*p*=0.0002) (**Figure 1A**). However, there was overlap with an area under receiver operating characteristic curve (auROC) of 86.7% (95% CI 77.7-95.7%).

### INSL3 in eugonadal men versus CHH

The median basal INSL3 was 1.08 ng/ml (0.95, 1.38) in eugonadal men and 0.05 ng/ml (0.01, 0.18) in men with CHH (*p*<0.0001) (**Figure 1B**). Whilst basal INSL3 could accurately differentiate all eugonadal men from those with CHH (auROC 100%, 95% CI 100-100%), it was unable to differentiate men with CHH according to their olfactory status (**Supplementary Figure 1**).

### INB in delayed puberty and eugonadal men versus CHH

**Figure 2:**
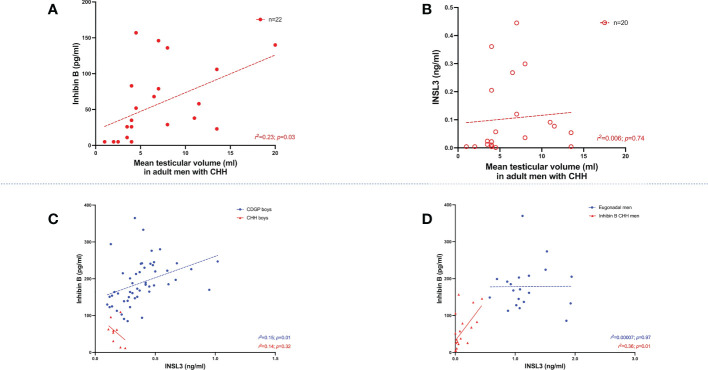
**(A)** Relationship between INB (pg/ml) and mean testicular volume (ml) in adult men with CHH (*n*=22). Simple linear regression *r* ^2 =^ 0.23; *p*=0.03. **(B)** Relationship between INSL3 (ng/ml) and mean testicular volume (ml) in adult men with CHH (*n*=20). Simple linear regression *r* ^2 =^ 0.006; *p*=0.74. **(C)** Relationship between basal INSL3 (ng/ml) levels over INB (pg/ml) in boys with CDGP (blue circles) and CHH (red triangles). Simple linear regression *r* ^2 =^ 0.15; *p*=0.01 in CDGP (blue dotted line). Simple linear regression *r* ^2 =^ 0.14; *p*=0.32 in CHH (red solid line). **(D)** Relationship between basal INSL3 (ng/ml) levels over INB (pg/ml) in eugonadal men (blue circles) and men with CHH (red triangles). Simple linear regression *r* ^2 =^ 0.00007; *p*=0.97 in eugonadal men (blue dotted line). Simple linear regression *r* ^2 =^ 0.36; *p*=0.01 in men with CHH (red solid line).

The median basal INB was 182 pg/ml (150, 230) in boys with CDGP and 59 pg/ml (22.5, 79.5) in boys with CHH (*p*<0.0001) (**Figure 1C**). When compared to INSL3, INB performed better in differentiating boys with CDGP from those with CHH (auROC 98.5%, 95% CI 95.9-100%). Median INB was 170 pg/ml (134, 205) in adult eugonadal men, and 36.5 pg/ml (9.5, 88.8) in adult men with CHH (*p*<0.0001) (**Figure 1D**). Thus, INB performed less well in differentiating eugonadal men from those with CHH (auROC 93.9%, 95% CI 87.2-100%) in comparison to INSL3. Basal INB levels significantly correlated with mean TV in men with CHH, this significant association with TV was not seen with INSL3, LH, FSH and testosterone (**Figures 2A, B**).

### Associations between INSL3 and INB

In boys with CDGP, there are significant positive associations between INSL3 and INB (*r^2 =^* 0.15, *p*=0.01) (**Figure 2C** **)**. The *r^2^* values is weak and thus this data should be interpreted in this context. Similarly, in adults with CHH there is also a significant positive association between INSL3 and INB, *r^2 =^* 0.36, *p*=0.01 (**Figure 2D** **)**. However, significant associations were not observed in boys with CHH, nor in adult eugonadal men.

### INSL3 and GnRHa/GnRH stimulated LH, FSH and testosterone

**Figure 3:**
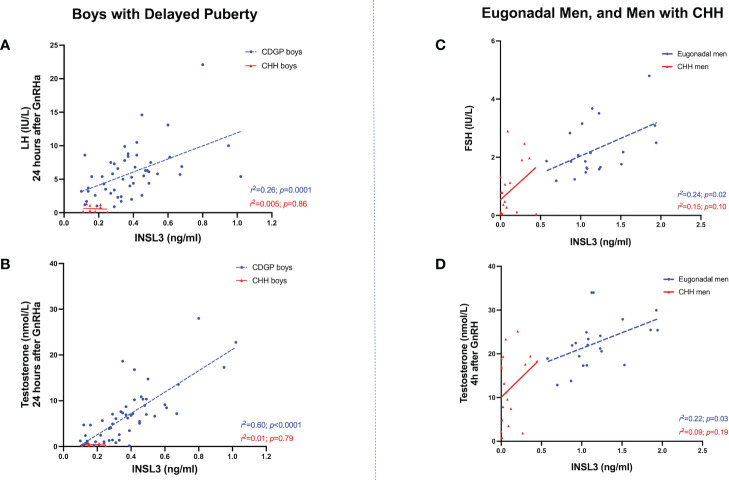
**(A)** Relationship between INSL3 (ng/ml) levels over stimulated LH (IU/L) at 24 hours after GnRHa in boys with CDGP (blue circles) and boys with CHH (red triangles). Simple linear regression *r* ^2 =^ 0.26; *p*=0.0001 in CDGP (blue dotted line); *r* ^2 =^ 0.005; *p*=0.86 in CHH (red solid line). **(B)** Relationship between INSL3 (ng/ml) levels over stimulated testosterone (nmol/L) at 24 hours after GnRHa in boys with CDGP (blue circles) and boys with CHH (red triangles). Simple linear regression *r* ^2 =^ 0.60; *p*<0.0001 in CDGP (blue dotted line); *r* ^2 =^ 0.01; *p*=0.79 in CHH (red solid line). **(C)** Relationship between INSL3 (ng/ml) levels over basal FSH (IU/L) in eugonadal men (blue circles) and men with CHH (red triangles). Simple linear regression *r* ^2 =^ 0.24; *p*=0.02 in eugonadal men (blue dotted line); *r* ^2 =^ 0.15; *p*=0.10 in CHH (red solid line). **(D)** Relationship between INSL3 (ng/ml) levels over stimulated testosterone (nmol/L) at 4 hours after GnRH in eugonadal men (blue circles) and men with CHH (red triangles). Simple linear regression *r* ^2 =^ 0.22; *p*=0.03 in eugonadal men (blue dotted line); *r* ^2 =^ 0.09; *p*=0.19 in CHH (red solid line).

In boys with CDGP, INSL3 positively correlated with GnRHa stimulated LH at 4 hours: *r^2 =^* 0.17, *p*=0.003; 24 hours: *r^2 =^* 0.26, *p*=0.0001 (see **Figure 3A** and **Table 1** **)**. As previous, the *r^2^* values is weak and thus this data should be interpreted in this context. INSL3 also correlated positively with basal and stimulated testosterone in CDGP (basal: *r^2 =^* 0.30, *p*<0.0001; 4 hour LH: *r^2 =^* 0.50, *p*<0.0001; 24 hour LH: *r^2 =^* 0.60, *p*<0.0001) (**Figure 3B** and **Table 1**). In eugonadal adult men, INSL3 was not associated with LH, but was positively related to baseline FSH levels (**Figure 3C** and **Table 1**), and to testosterone levels at 4 hours after GnRH (**Figure 3D** and **Table 1**).

**Table 1:** Linear regression analysis of INSL3 vs baseline and stimulated LH, FSH, testosterone in the delayed puberty and adult cohorts.

	*Boys with CHH*			*Boys with CDGP*				*Men with CHH*			*Eugonadal men*		
INSL3 versus							INSL3 versus						
	*r*	*r* ^2^	*P*-value	*r*	*r* ^2^	*P*-value		*r*	*r* ^2^	*P*-value	*r*	*r* ^2^	*P*-value
Baseline LH (IU/L)	0.24	0.11	0.39	1.205	0.07	0.05	Baseline LH (IU/L)	1.78	0.17	0.07	0.56	0.04	0.38
4hr LH (IU/L) (GnRHa)	-2.76	0.005	0.85	**23.48**	**0.17**	**0.003**	30min LH (IU/L) (GnRH)	12.69	0.03	0.48	4.54	0.03	0.47
24hr LH (IU/L) (GnRHa)	-0.75	0.005	0.86	**9.75**	**0.26**	**0.0001**	4hr LH (IU/L) (GnRH)	6.90	0.07	0.25	1.16	0.03	0.45
Baseline FSH (IU/L)	2.74	0.23	0.19	1.26	0.06	0.09	Baseline FSH (IU/L)	2.51	0.15	0.10	**1.21**	**0.24**	**0.02**
4hr FSH (IU/L) (GnRHa)	-3.92	0.003	0.88	-5.9	0.04	0.14	30min FSH (IU/L) (GnRH)	3.83	0.03	0.46	2.63	0.16	0.08
24hr FSH (IU/L) (GnRHa)	-10.93	0.21	0.22	-1.26	0.005	0.61	4hr FSH (IU/L) (GnRH)	3.78	0.04	0.40	1.93	0.16	0.08
Baseline T (nmol/L)	0.80	0.03	0.65	**4.75**	**0.30**	**<0.0001**	Baseline T (nmol/L)	16.26	0.09	0.19	3.74	0.04	0.36
4hr T (nmol/L) (GnRHa)	-0.49	0.02	0.72	**9.47**	**0.50**	**<0.0001**	30min T (nmol/L) (GnRH)	17.86	0.12	0.13	4.71	0.08	0.23
24hr T (nmol/L) (GnRHa)	-0.47	0.01	0.79	**23.33**	**0.60**	**<0.0001**	4hr T (nmol/L) (GnRH)	18.03	0.09	0.19	**7.16**	**0.22**	**0.03**

Statistically significant results are highlighted in bold. The regression coefficient r is presented, as well as r^2^ and p-value after linear regression. CHH, congenital hypogonadotrophic hypogonadism; CDGP, constitutional delay of growth and puberty; LH, luteinising hormone; FSH, follicle stimulating hormone; T, testosterone; GnRH, gonadotrophin releasing hormone; GnRHa, gonadotrophin releasing hormone agonist.

### INB and GnRHa/GnRH stimulated LH, FSH and testosterone

**Figure 4:**
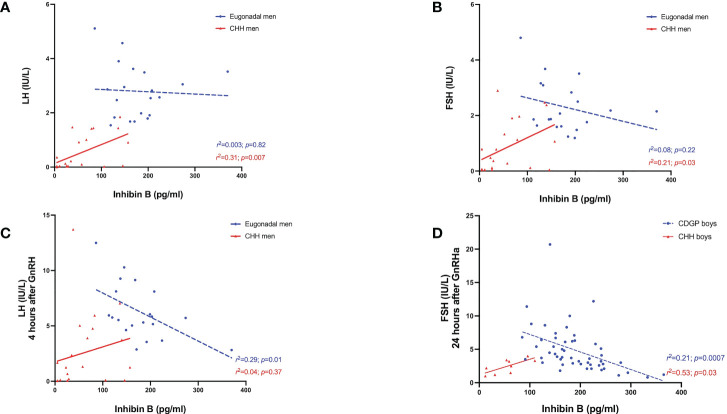
**(A)** Relationship between INB (pg/ml) levels over basal LH (IU/L) in eugonadal men (blue circles) and men with CHH (red triangles). Simple linear regression *r* ^2 =^ 0.003; *p*=0.82 in eugonadal men (blue dotted line); *r* ^2 =^ 0.31; *p*=0.007 in men with CHH (red solid line). **(B)** Relationship between INB (pg/ml) levels over basal FSH (IU/L) in eugonadal men (blue circles) and men with CHH (red triangles). Simple linear regression *r* ^2 =^ 0.08; *p*=0.22 in eugonadal men (blue dotted line); *r* ^2 =^ 0.21; *p*=0.03 in men with CHH (red solid line). **(C)** Relationship between INB (pg/ml) levels over stimulated LH (IU/L) 4 hours after GnRH in eugonadal men (blue circles) and men with CHH (red triangles). Simple linear regression *r* ^2 =^ 0.29; *p*=0.01 in eugonadal men (blue dotted line); *r* ^2 =^ 0.04; *p*=0.37 in men with CHH (red solid line). **(D)** Relationship between INB (pg/ml) levels over stimulated FSH (IU/L) 24 hours after GnRHa in boys with CDGP (blue circles) and boys with CHH (red triangles). Simple linear regression *r* ^2 =^ 0.21; *p*=0.0007 in boys with CDGP (blue dotted line); *r* ^2 =^ 0.53; *p*=0.03 in boys with CHH (red solid line).

In delayed puberty, no significant associations were found between INB and either basal or stimulated LH (**Table 2**). In adult men with CHH, INB was positively related to both baseline LH (*r^2 =^* 0.31, *p*=0.007) (**Figure 4A**) and FSH (*r^2 =^* 0.21, *p*=0.03) (**Figure 4B**), whereas in eugonadal men, INB was negatively associated with LH levels at 4 hours after GnRH (*r^2 =^* 0.29, *p*=0.01) (**Figure 4C**). INB was positively related to FSH at 24 hours after GnRHa in boys with CHH (*r^2 =^* 0.53, *p*=0.03), but negatively related in boys with CDGP (4 hours: *r^2 =^* 0.35, *p*<0.0001; 24 hours: *r^2 =^* 0.21, *p*=0.0007) (**Figure 4D**). There were significant positive associations between INB and testosterone in CDGP (basal testosterone: *r^2 =^* 0.10 *p*=0.02 and 24 hour testosterone: *r^2 =^* 0.09, *p*=0.03), but these associations were not significant in boys with CHH or in adult men (**Table 2**).

**Table 2:** Linear regression analysis of INB versus baseline and stimulated LH, FSH, testosterone in the delayed puberty and adult cohorts.

	*Boys with CHH*			*Boys with CDGP*				*Men with CHH*			*Eugonadal men*		
INB versus							INB versus						
	*r*	*r* ^2^	*P*-value	*r*	*r* ^2^	*P*-value		*r*	*r* ^2^	*P*-value	*r*	*r* ^2^	*P*-value
Baseline LH (IU/L)	0.0006	0.37	0.08	0.002	0.02	0.31	Baseline LH (IU/L)	**0.007**	**0.31**	**0.007**	-0.0008	0.003	0.82
4hr LH (IU/L) (GnRHa)	0.02	0.21	0.22	-0.004	0.0005	0.88	30min LH (IU/L) (GnRH)	0.04	0.03	0.44	-0.07	0.19	0.05
24hr LH (IU/L) (GnRHa)	0.009	0.37	0.08	0.003	0.002	0.76	4hr LH (IU/L) (GnRH)	0.01	0.04	0.37	**-0.02**	**0.29**	**0.01**
Baseline FSH (IU/L)	**-0.005**	**0.08**	**0.05**	0.003	0.15	0.30	Baseline FSH (IU/L)	**0.008**	**0.21**	**0.03**	-0.004	0.08	0.22
4hr FSH (IU/L) (GnRHa)	0.04	0.22	0.20	**-0.05**	**0.35**	**<0.0001**	30min FSH (IU/L) (GnRH)	0.008	0.02	0.55	-0.02	0.17	0.07
24hr FSH (IU/L) (GnRHa)	**0.02**	**0.53**	**0.03**	**-0.03**	**0.21**	**0.0007**	4hr FSH (IU/L) (GnRH)	0.007	0.02	0.52	-0.01	0.15	0.10
Baseline T (nmol/L)	0.0007	0.01	0.78	**0.009**	**0.10**	**0.02**	Baseline T (nmol/L)	0.05	0.16	0.07	-0.01	0.01	0.67
4hr T (nmol/L) (GnRHa)	0.002	0.17	0.26	0.01	0.06	0.08	30min T (nmol/L) (GnRH)	0.04	0.10	0.17	0.02	0.05	0.35
24hr T (nmol/L) (GnRHa)	0.002	0.07	0.50	**0.03**	**0.09**	**0.03**	4hr T (nmol/L) (GnRH)	0.04	0.08	0.23	0.01	0.01	0.62

Statistically significant results are highlighted in bold. The regression coefficient r is presented, as well as r^2^ and p-value after linear regression. CHH, congenital hypogonadotrophic hypogonadism; CDGP, constitutional delay of growth and puberty; LH, luteinising hormone; FSH, follicle stimulating hormone; T, testosterone; GnRH, gonadotrophin releasing hormone; GnRHa, gonadotrophin releasing hormone agonist.

## Discussion

We found that boys with delayed puberty due to CHH had lower INSL3 levels than boys with CDGP, however there were some overlap in the INSL3 levels between boys with CDGP and CHH (auROC of 86.7%). In comparison, all eugonadal adult men had higher INSL3 levels than adult men with CHH and there was no overlap in INSL3 levels with an auROC of 100%.

Basal INSL3 was not able to differentiate CHH men according to their olfactory status or the presence of pathogenic or likely pathogenic variants. In contrast, LH responses to kisspeptin-54 were even lower in those with anosmia or pathogenic variants identified in comparison to other men with CHH (28), suggesting that kisspeptin can provide functional assessment in patients with CHH. Additionally, the LH response to kisspeptin-10 could provide prognostic classification of subsequent pubertal development in boys and girls with delayed puberty (30). Additional prospective studies in larger cohorts are needed to realise this potential for translation to clinical practice.

INB was higher in boys with CDGP compared to those with CHH with an auROC of 98.5%, and likewise higher in eugonadal adult men compared to those with CHH (auROC of 93.9%). In boys with CDGP, significant positive associations between INSL3, INB, and testosterone were observed, likely reflecting the positive relationship between Leydig and Sertoli cell populations and functionality during pubertal progression. Interestingly, in adult men with CHH, TV positively correlated with INB, but not with INSL3. This stands to reason, as after puberty, INB production is directly proportional to spermatogenic status and germ cell mass in addition to Sertoli cells mass, which together comprise the majority of TV (11).

These data are consistent with INSL3 being more reflective of the attainment of complete pubertal development, whereas INB appears to have greater predictive power in the setting of boys with delayed puberty. One reason for this could be that INSL3 changes more slowly in comparison to INB. As previously reported in cell culture studies, INSL3 is largely a constitutive secretory product of Leydig cells and is not acutely regulated in the short-term (hours) by gonadotrophins (26). In contrast, increments in INSL3 production due to Leydig cell proliferation and differentiation following gonadotrophin exposure takes place over several days and is a chronic differentiation dependent process (26). This is evidenced by a report in healthy adult men who were acutely exposed to supraphysiological levels of LH-like bioactivity following which an acute rise in serum testosterone was seen but not in INSL3 (31). In contrast, a chronic gonadotrophin/hCG stimulus over a period of weeks to months did result in a change in INSL3 (25, 32). Thus, although INSL3 production and secretion is dependent on LH, INSL3 is a longer-term signal of the trophic effect of LH on Leydig cell structure and function (25). Overall, INSL3 could represents an additional downstream marker of the Leydig cell function when assessing the HPG axis (26).

Interestingly in boys with CDGP, there were positive associations between INSL3, LH and testosterone (basal and following GnRHa stimulation), however such associations were not seen in the eugonadal men or both CHH cohorts. This may reflect the spectrum of maturing Leydig cells present in boys with CDGP thus demonstrating variable incremental LH response to GnRHa as boys traverse the different stages of puberty (23). In contrast in adult eugonadal men, the mature Leydig cell population could already be operating at ‘functional capacity’ and thus these associations were no longer evident. Another plausible explanation for this difference is that GnRHa was used in the paediatric cohort, whilst GnRH was used in the adult cohort. As GnRHa have higher receptor affinity and longer half-lives than GnRH (33), the resultant supraphysiological and sustained stimulation of the gonadotrophic cells may lead to greater LH releases which could better reveal correlations with INSL3.

Boys with CDGP and eugonadal adult men demonstrated negative associations between INB, and basal and stimulated LH as well as FSH levels. In contrast in both boys and men with CHH, positive associations between INB and gonadotrophins were observed. The reason for this discrepancy is intriguing, but perhaps some element of endocrine feedback is present in boys with CDGP and eugonadal adult men that is yet to be established in the CHH cohort.

To date there are limited studies of INSL3 in the context of CDGP and no previous studies have compared the performance of INSL3 and INB. This is the first study to explore INSL3 levels in both a paediatric and an adult cohort of CHH comparing these to CDGP and eugonadal control respectively. It confirms previous findings of lower INSL3 levels in a CHH cohort compared to eugonadal adults (25), however another report showed no significant difference between INSL3 in CHH and prepubertal CDGP cohorts (16).

Limitations of this study are its retrospective analysis of previous cohorts and the small numbers of patients with CHH, which is a rare condition. The adult men cohort with CHH were also significantly older than the adult eugonadal cohort and were examined after a pause in testosterone treatment. Some but not all of the men with CHH have had previous gonadotrophin treatments, with duration of treatment ranging from 6 months to 5 years. Although study visits were conducted after a washout period in adult men with CHH, it remains possible that the different treatments received could have impacted on the measurements taken in the study. The paediatric cohort were followed up to 24 months after assessment. A diagnosis of CDGP was made if the TV reached ≥8ml within 12-18 months of follow-up, and of CHH if TV did not reach ≥5ml within 24 months of follow-up. Thus, it is possible that some patients assigned as CDGP could have subsequently had arrested pubertal development following discharge, although none of this cohort re-presented for further assessment. The auROC in this study is calculated using data from the adult and paediatric cohorts included and there is a need for validation in external cohorts.

At present there is no ‘gold standard’ diagnostic test that unequivocally differentiates CDGP and CHH (34). Previously described diagnostic tests include measurement of unstimulated basal/nocturnal gonadotrophins (35, 36), and stimulated gonadotrophin levels following GnRH, GnRHa, or human chorionic gonadotrophin (hCG) (10). Unfortunately, the limited sensitivity, specificity, complexity, and invasiveness of previously described stimulation protocols has limited universal adoption for the assessment of delayed puberty (10, 34).

A recent study published findings of GnRH stimulation test and INB in a large cohort of men with CHH (*n*=127), CDGP (*n*=74), and healthy controls (*n*=31) (37). There were variable LH responses to the GnRH test, with 47% of patients with CHH having peak LH levels that overlapped with those in the CDGP group (37). For INB, 50% of levels overlapped between those with CDGP and those with CHH (37). Consistent with the findings in the present study, positive correlation between INB and TV was described (37).

Binder et al. have also reported the accuracy of a composite outcome based on both the LH response to GnRHa and INB in prepubertal males aged 13.7-17.5 years (27), which provided 100% sensitivity and 98.1% specificity when LH <0.3 IU/L and INB <111 pg/ml cut-offs were applied (27). More recently in addition to kisspeptin-stimulated-LH (30) (discussed above), FSH-stimulated-INB (38) and GnRHa-stimulated INB (39) have been proposed as potential diagnostic tests to predict outcomes in delayed puberty.

Recently liquid chromatography-tandem mass spectrometry (LC-MS/MS) has been validated for the quantification of INSL3 (17) and reference range established and tested in healthy boys progressing through puberty (18), males with hypogonadotrophic hypogonadism and Klinefelter syndrome (40). Using this assay, INSL3 concentrations were lower in both untreated boys with Klinefelter syndrome (*n*=83) or hypogonadotrophic hypogonadism (*n*=103). Unlike serum testosterone, INSL3 was not affected by BMI, body fat percentage or alcohol consumption (41) and was not affected by diurnal variation.

In summary, INSL3 appears to be able to accurately identify the transition to the post-pubertal adult male state, whereas INB may have more predictive capability in boys with CDGP. This suggests that INSL3 could be used to monitor response to treatment in boys with delayed puberty as they transition towards adulthood pending further longitudinal study.

## Data availability statement

The raw data supporting the conclusions of this article will be made available by the authors, without undue reservation.

## Ethics statement

The studies involving human participants were reviewed and approved by West London Research Ethics Committee, London, United Kingdom (UK) (reference: 12/LO/0507) and Ethical Committee of the Medical Faculty of the University of Tübingen, Germany. Written informed consent to participate in this study was provided by the participants’ legal guardian/next of kin.

## Author contributions

All authors provided contributions to study conception and design, acquisition of data or analysis and interpretation of data, drafting the article or revising it critically for important intellectual content, and final approval of the version to be published. Here are the most important contributions of each author: AA, GB, RA-I, RI, WD designed the study. AA, KK, MP, PE, SC, AC, LY, CI-E and GB conducted experiments and acquired the data. AA, MP, PE, SC, NS, CJ, RQ and GB recruited patients for the clinical studies. AA, KK, MP, PE, SH, CX, NP, RA-I and RI analysed the data. AA, MP, PE, CX, NP, GB, RA-I and RI conducted assays for biochemical analytes;. and all authors contributed to the writing and revision of the manuscript. RI and WD take final responsibility for this article.

## Funding

The study was designed, conducted, analysed, and reported entirely by the authors. This paper presents independent research funded by grants from the NIHR and supported by the NIHR/Wellcome Trust Imperial Clinical Research Facility and NIHR Imperial Biomedical Research Centre. The Section of Endocrinology and Investigative Medicine is funded by grants from the MRC and NIHR. The views expressed are those of the author(s) and not necessarily those of the MRC, the NHS, the NIHR, or the Department of Health. AA was supported by National Institute of Health Research (NIHR) Clinician Scientist Award CS-2018-18-ST2-002. KK was supported by NIHR Academic Clinical Fellowship Award ACF-2021-21-001 and acknowledges infrastructure support for this research from the NIHR Imperial Biomedical Research Centre (BRC). MP was supported by an NIHR Academic Clinical Lectureship Award. CI-E was supported by an Imperial-BRC IPPRF Award (P79696). SH was supported by the UKRI CDT in AI for Healthcare http://ai4health.io (Grant number EP/S023283/1). GB, RA-I, and RI were supported by institutional funds and are grateful to Ms Yanzhenzi Dai and Karin Weber for technical support. WD was supported by an NIHR Research Professorship NIHR-RP-2014-05-001 and NIHR Senior Investigator Award.

## Conflict of interest

AA has undertaken consultancy work for Myovant Sciences Ltd. WD has undertaken consultancy work for Myovant Sciences Ltd and KaNDy Therapeutics.

The remaining authors declare that the research was conducted in the absence of any commercial or financial relationships that could be construed as a potential conflict of interest.

## Publisher’s note

All claims expressed in this article are solely those of the authors and do not necessarily represent those of their affiliated organizations, or those of the publisher, the editors and the reviewers. Any product that may be evaluated in this article, or claim that may be made by its manufacturer, is not guaranteed or endorsed by the publisher.
