# Supplementary material for: Insulin-like peptide 3 (INSL3) in congenital hypogonadotrophic hypogonadism (CHH) in boys with delayed puberty and adult men
Source: Front Endocrinol (Lausanne). 2022 Nov 29;13:1076984. doi: 10.3389/fendo.2022.1076984 (PMC9745113; doi:10.3389/fendo.2022.1076984)
Supplement: Supplementary file 1 [file DataSheet_1.docx]

**
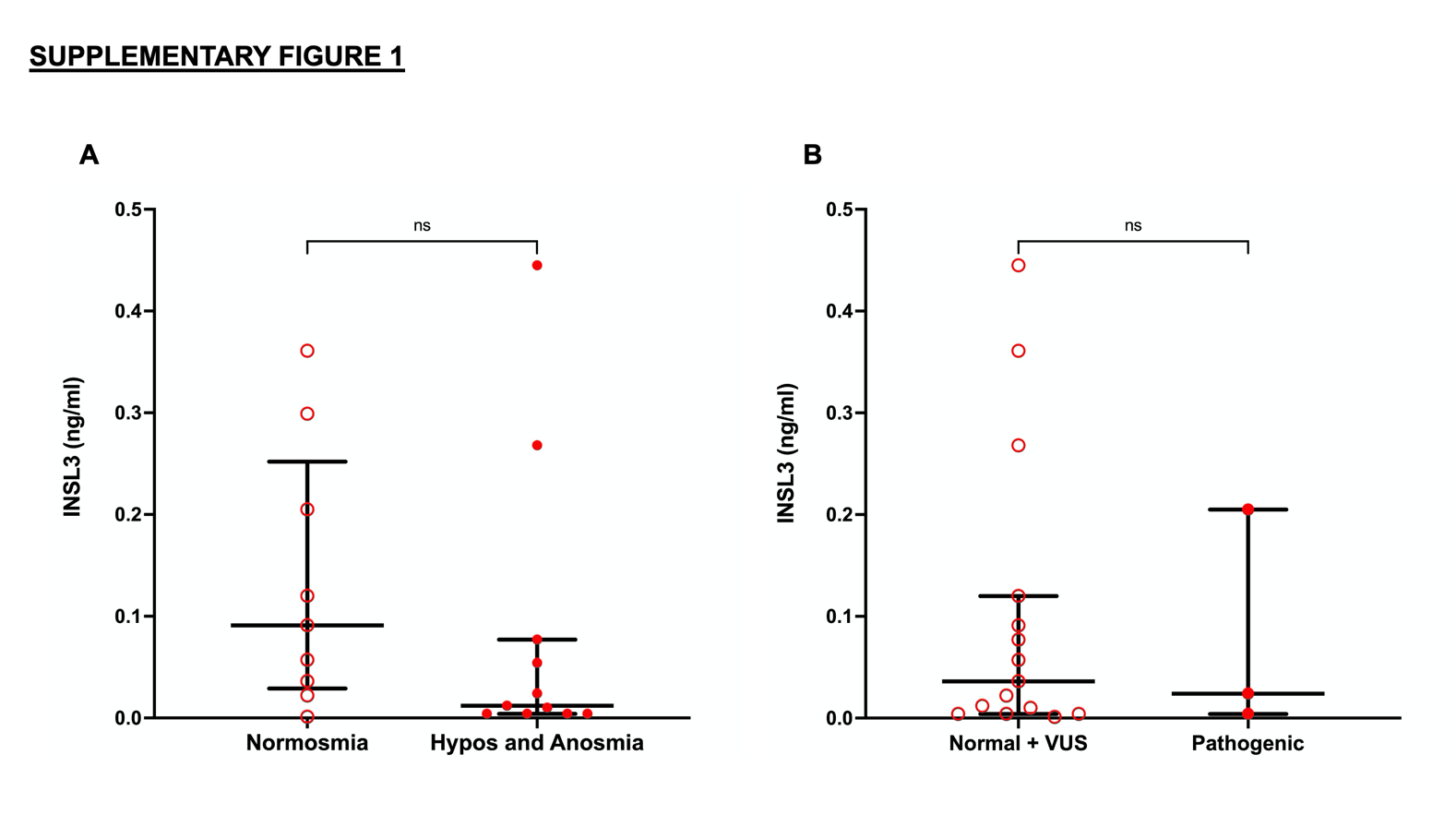
**

**Supplementary Figure 1:**

A. Scattergram (median, IQR) of basal INSL3 (ng/ml) in CHH men grouped by their olfactory status – normosmia, hyposmia and anosmia determined by the 40-item University of Pennsylvania Smell Identification Test (UP-SIT). Groups were compared using the Mann-Whitney U test.

B. Scattergram (median, IQR) of basal INSL3 (ng/ml) in CHH men grouped by those with pathogenic/likely pathogenic variants compared to those with VUS or no abnormalities detected on genetic testing. Groups were compared using the Mann-Whitney U test.

VUS = variant of unknown significance.
