# Supplementary material for: Insulin-like peptide 3 (INSL3) in congenital hypogonadotrophic hypogonadism (CHH) in boys with delayed puberty and adult men
Source: Front Endocrinol (Lausanne). 2022 Nov 29;13:1076984. doi: 10.3389/fendo.2022.1076984 (PMC9745113; doi:10.3389/fendo.2022.1076984)
Supplement: Supplementary file 2 [file Table_1.docx]

**Supplementary Table 1. Clinical characteristics at first study visit in boys with CDGP or CHH and in eugonadal men or men with CHH**

| **Clinical characteristic** | **Delayed Puberty:**  **CDGP**  **(n=51)** | **Delayed Puberty:**  **CHH**  **(n=9)** | ***P*-value** | **Adult:**  **Eugonadal men**  **(*n*=22)** | **Adult:**  **Men with CHH**  **(*n*=22)** | ***P*-value** |
| --- | --- | --- | --- | --- | --- | --- |
| **Age (years)** | 14.9 ± 0.8 | 14.6 ± 1.0 | 0.270 | 24.1 ± 4.53 | 38.4 ± 13.7 | 0.0002 |
| **BMI (kg/m^2^)** | NA | NA | NA | 23 ± 2.27 | 26.4 ± 5.03 | 0.0219 |
| **BMI, SDS_LMS_^*^** | -0.9 ± 1.5 | 0.8 ± 1.4 | 0.003 | NA | NA | NA |
| **Bone age retardation (years)**** | 2.3 ± 0.8 | 1.4 ± 0.9 | 0.004 | NA | NA | NA |
| **Testicular volume (ml)** | 3.1 ± 0.9 | 1.6 ± 0.4 | <0.001 | NA | 4.50 (3.88, 8.75) | NA |
| **Serum INSL3 (ng/ml)** | 0.35 (0.24, 0.47) | 0.15 (0.14, 0.21) | 0.0002 | 1.08 (0.95, 1.38)^†^ | 0.05 (0.01, 0.18)^†^ | <0.0001 |
| **Serum INB (pg/ml)** | 182 (150, 230) | 59 (22.5, 79.5) | <0.0001 | 170 (134, 205)^††^ | 36.5 (9.50, 88.8) | <0.0001 |
| **Serum LH (IU/L)** | 0.80 (0.50, 1.40) | 0.10 (0.10, 0.10) | <0.0001 | 2.84 (1.89, 3.69) | 0.12 (0.04, 1.11) | <0.0001 |
| **Serum FSH (IU/L)** | 2.10 (1.30, 2.70) | 0.40 (0.30, 0.80) | <0.0001 | 1.97 (1.63, 2.90) | 0.43 (0.07, 1.48) | <0.0001 |
| **Serum Testosterone**  **(nmol/L)** | 0.69 (0.23, 2.05) | 0.23 (0.14, 0.46) | 0.027 | 19.6 (15.0, 25.6) | 11.7 (4.3, 16.8) | <0.0001 |

Full data on background characteristics have previously been published in (23,24). A brief summary of key characteristics is presented.

Mean ± SD is presented for parametrically distributed data and median (25^th^ centile, 75^th^ centile) is presented for non-parametrically distributed data. Parametrically distributed data were compared using the unpaired *t* test and non-parametrically distributed data by the Mann-Whitney U test.

CHH, congenital hypogonadotrophic hypogonadism; INSL3, insulin-like peptide 3; INB, inhibin-B; LH, luteinising hormone; FSH, follicle-stimulating hormone; SD, standard deviation; NA, not applicable/measured.

*BMI in the delayed puberty cohort were reported in standard deviation score (SDS_LMS_) values according to Kromeyer-Hauschild *et al* (36)

**Bone age retardation = chronological age MINUS bone age

^†^*n*=21 in adult eugonadal men and *n*=20 in adult men with CHH for INSL3 due to missing data.

^††^*n*=20 in adult eugonadal men for INB due to missing data
